# High Sugar Intake and Development of Skeletal Muscle Insulin Resistance and Inflammation in Mice: A Protective Role for PPAR-**δ** Agonism

**DOI:** 10.1155/2013/509502

**Published:** 2013-06-18

**Authors:** Elisa Benetti, Raffaella Mastrocola, Mara Rogazzo, Fausto Chiazza, Manuela Aragno, Roberto Fantozzi, Massimo Collino, Marco A. Minetto

**Affiliations:** ^1^Department of Drug Science and Technology, University of Turin, Via Giuria 9, 10125 Torino, Italy; ^2^Department of Clinical and Biological Sciences, University of Turin, Corso Raffaello 30, 10125 Torino, Italy; ^3^Division of Endocrinology, Diabetology and Metabolism, Department of Medical Sciences, University of Turin, Corso Dogliotti 14, 10126 Torino, Italy

## Abstract

Peroxisome Proliferator Activated Receptor (PPAR)-**δ** agonists may serve for treating metabolic diseases. However, the effects of PPAR-**δ** agonism within the skeletal muscle, which plays a key role in whole-body glucose metabolism, remain unclear. This study aimed to investigate the signaling pathways activated in the gastrocnemius muscle by chronic administration of the selective PPAR-**δ** agonist, GW0742 (1 mg/kg/day for 16 weeks), in male C57Bl6/J mice treated for 30 weeks with high-fructose corn syrup (HFCS), the major sweetener in foods and soft-drinks (15% wt/vol in drinking water). Mice fed with the HFCS diet exhibited hyperlipidemia, hyperinsulinemia, hyperleptinemia, and hypoadiponectinemia. In the gastrocnemius muscle, HFCS impaired insulin and AMP-activated protein kinase signaling pathways and reduced GLUT-4 and GLUT-5 expression and membrane translocation. GW0742 administration induced PPAR-**δ** upregulation and improvement in glucose and lipid metabolism. Diet-induced activation of nuclear factor-*κ*B and expression of inducible-nitric-oxide-synthase and intercellular-adhesion-molecule-1 were attenuated by drug treatment. These effects were accompanied by reduction in the serum concentration of interleukin-6 and increase in muscular expression of fibroblast growth factor-21. Overall, here we show that PPAR-**δ** activation protects the skeletal muscle against the metabolic abnormalities caused by chronic HFCS exposure by affecting multiple levels of the insulin and inflammatory cascades.

## 1. Introduction

In healthy humans, skeletal muscle accounts for ~70–80% of the insulin-stimulated glucose uptake, being the major site of glucose disposal and, thus, exerting a key role in regulating whole body glucose homeostasis. Accordingly, understanding changes that occur to this tissue during obesity and diabetes development is crucial to elucidate the underlying causes of insulin resistance and to reveal new targets for its treatment. Insulin resistance in skeletal muscle has long been recognized as a characteristic feature of type 2 diabetes and plays a major role in the pathogenesis of the disease [1]. Although several epidemiological data have shown that the consumption of added sugars as ingredients in processed or prepared foods and caloric beverages has dramatically increased over the last decades, most of the experimental studies investigating the development of insulin resistance in the skeletal muscle have been based on genetic manipulation or use of high fat diets [2–4]. In contrast, the molecular mechanisms underlying the detrimental effects of sugar, mainly those on skeletal muscle, are not completely understood. In the present study, we used a previously developed mouse model of chronic exposure to high-fructose corn syrup (HFCS) [5] to investigate the deleterious effects of high sugar intake on skeletal muscle. HFCS syrup, used as an ingredient in processed or prepared foods and caloric beverages, is synthesized by refining corn starch, contains 55% fructose and 42% glucose and to date accounts for over 40% of all added caloric sweeteners [6]. 

The Peroxisome Proliferator Activated Receptor (PPAR) superfamily of transcription factors, that includes the isoforms PPAR-*α*, PPAR-*δ*, and PPAR-*γ*, has been widely shown to exert crucial roles in energy homeostasis regulation and glucose metabolism. PPAR isoforms display tissue-specific expression and gene-regulatory profiles. PPAR-*δ*, one of the most promising pharmacological target implicated in obesity-associated insulin resistance [7], is highly expressed in skeletal muscle, at 10- and 50-folds higher levels compared with PPAR-*α* and PPAR-*γ*, respectively [8]. However, its potential effects in affecting skeletal muscle glucose intake and insulin sensitivity are only now being elucidated. Schuler et al. [9] showed that mice in which PPAR-*δ* is selectively ablated in skeletal myocytes exhibit fiber-type switching, obesity, and type 2 diabetes. Besides, a very recent paper has suggested that the improvement of glucose homeostasis by angiotensin receptor blockers in hypertensive patients involves a selective PPAR-*δ* activation in the skeletal muscle [10]. So far, the exact molecular mechanisms underlying the observed PPAR-*δ*-induced changes have not yet been determined. Hence, the present study aimed to determine whether chronic administration of the selective PPAR-*δ* agonist GW0742 in HFCS-fed mice may ameliorate the impairment of signaling pathways triggered in skeletal muscles by chronic high sugar intake. Several studies have revealed that a large number of muscle-derived secretory cytokines (collectively termed myokines) can act locally in an autocrine/paracrine manner, linking skeletal muscle to regulation of physiological processes in other tissues. We have, thus, also investigated whether PPAR-*δ* agonism may affect the serum levels of the well-known myokine interleukin-6 (IL-6) and the muscular expression of a member of the fibroblast growth factor (FGF) superfamily, FGF-21, a recently identified myokine involved in the interorgan communication [11]. To further extend our investigation on the ability of PPAR-*δ* agonism to modulate inflammatory pathways involved in local insulin resistance pathogenesis, the effects of PPAR-*δ* activation on nuclear translocation of the transcription nuclear factor-kappaB (NF-*κ*B) and expression of its target genes have also been studied. 

## 2. Materials and Methods

### 2.1. Animals and Diets

Four-week-old male C57Bl6/J mice (Harlan-Italy; Udine, Italy) were housed in a controlled environment at 25 ± 2°C with alternating 12-h light and dark cycles. They were provided with a Piccioni pellet diet (n. 48, Gessate Milanese, Italy) and water *ad libitum*. All the animals were fed with a normal pellet diet for 1 week prior to the experiment. The animals were then allocated to two dietary regimens, chow diet and normal drinking water (control) or a chow diet and 15% (wt/vol) HFCS solution in drinking water (HFCS) for 30 weeks. All diets contained a standard mineral and vitamin mixture. The concentration of HFCS solution as well as the period of dietary manipulation was chosen according to previous animal studies investigating the metabolic effects of long-term (6-7 months) access to HFCS. Body mass, intake of water, and food were recorded weekly. Animal care was in compliance with Italian regulations on the protection of animals used for experimental and other scientific purposes (DM 116/92), and the experiment was approved by the Turin University Ethics Committee.

### 2.2. Drug Administration

After the initial period of 14 weeks of dietary manipulation, each of the two diet groups (control and HFCS diet) was further subdivided to obtain four different treatment groups: chow diet and normal drinking water (control, *n* = 10), chow diet supplemented with GW0742 (1 mg/kg/day) and normal drinking water (control + GW, *n* = 6), chow diet and 15% (wt/vol) HFCS solution in drinking water (HFCS, *n* = 10), and chow diet supplemented with GW0742 (1 mg/kg/day) and 15% (wt/vol) HFCS solution in drinking water (HFCS + GW, *n* = 10). The drug was daily administered with the food for the last 16 weeks, and the mice were allowed to continue to feed on their respective diets until the end of the study. GW0742 is a highly potent and selective PPAR-*δ* agonist (murine EC_50_: 28 nM for PPAR-*δ*; 8,900 nM for PPAR-*α*; >10,000 nM for PPAR-*γ*), with an acceptable pharmacokinetic profile and activity *in vivo* [12]. The dose and the kinetics of administration were chosen based on those we have previously shown to improve glucose tolerance and insulin sensitivity *in vivo* [5]. 

### 2.3. Oral Glucose Tolerance Test (OGTT)

One day before the mice were due to be killed, the OGTT was performed after a fasting period of 6 h by administering glucose (2 g/kg) by oral gavage. Once before administration and 15, 30, 60, 90, 120, and 150 min afterward, blood was obtained from the saphenous vein, and glucose concentration was measured with a conventional Glucometer (Accu-Check Compact kit, Roche Diagnostics Gmbh, Mannheim, Germany). 

### 2.4. Blood Biochemical Analysis

After 16 weeks from the start of the drug treatment (i.e., after 30 weeks of dietary manipulation), the mice were anaesthetised with i.p. injection (30 mg/kg) of Zoletil 100 (Laboratories Virbac, France) and killed by aortic exsanguination. Blood samples were collected, and plasma was isolated. Glycemia was measured using the Accu-Check Compact kit. The serum lipid profile was determined by measuring the content of triglycerides, total cholesterol, high-density lipoprotein (HDL), and low density lipoprotein (LDL) by standard enzymatic procedures using reagent kits (Hospitex Diagnostics, Florence, Italy). Plasma leptin and adiponectin levels were measured using enzyme-linked immunosorbent assay (ELISA) kits (Leptin and Adiponectin Mouse ELISA Kits, Abcam, Cambridge UK). The gastrocnemius muscle and epididymal fat were isolated, weighed, rapidly freeze-clamped with liquid nitrogen, and stored at −80°C.

### 2.5. Tissue Extracts

Gastrocnemius extracts were prepared using the Meldrum method [13] with modification. Briefly, gastrocnemius were homogenised at 10%(w/v) in a Potter-Elvehjem homogenizer (Wheaton, NJ, USA) using a homogenisation buffer containing 20 mM HEPES, pH 7.9, 1 mM MgCl_2_, 0.5 mM EDTA, 1 mM EGTA, 1 mM dithiothreitol (DTT), 0.5 mM phenylmethylsulfonyl fluoride (PMSF), 5 *μ*g/mL aprotinin, and 2.5 *μ*g/mL leupeptin. Homogenates were centrifuged at 4000 RPM at 4°C for 5 min. Supernatants were removed and centrifuged at 14000 RPM at 4°C for 40 minutes to obtain the cytosolic fraction. The pelleted nuclei were resuspended in an extraction buffer and centrifuged at 14000 RPM for 20 minutes at 4°C. The supernatants thus obtained, containing nuclear proteins, were carefully removed. The amount of proteins contained in the cytosolic and nuclear fraction was determined using a BCA protein assay following the manufacturers' instructions. Samples were stored at −80°C until use.

### 2.6. Skeletal Muscle Triglyceride Level

Skeletal muscle triglycerides were extracted from gastrocnemius homogenates and assayed using reagent kits according to the manufacturer's instructions (Triglyceride Quantification Kit, Abnova Corporation, Aachen, Germany).

### 2.7. Western Blot Analysis

About 60 *μ*g of total proteins was loaded for western blot experiments. Proteins were separated by 8% sodium dodecyl sulphate-polyacrylamide gel electrophoresis (SDS-PAGE) and transferred to a polyvinyldene difluoride (PVDF) membrane, which was then incubated with a primary antibody (rabbit anti-PPAR-*δ*, dilution 1 : 500; rabbit antitotal GSK-3*β*, dilution 1 : 200; goat anti-pGSK-3*β* Ser^9^, dilution 1 : 200; rabbit antitotal Akt, dilution 1 : 1000; mouse anti-pAkt Ser^473^, dilution 1 : 1000; goat anti-ICAM-1, dilution 1 : 500; rabbit antitotal IRS-1, dilution 1 : 200; goat anti-pIRS-1 Ser^307^, dilution 1 : 200; rabbit anti-GLUT-4, dilution 1 : 2000; rabbit anti-GLUT-5, dilution 1 : 100; rabbit anti-iNOS, dilution 1 : 200; rabbit anti-NF-*κ*B dilution 1 : 1000; rabbit antitotal AMPK, dilution 1 : 1000; rabbit anti-pAMPK Thr^172^, dilution 1 : 1000; rabbit antitotal ACC, dilution 1 : 500; rabbit anti-pACC Ser^79^, dilution 1 : 1000; rabbit anti-CPT-1, dilution 1 : 200). Blots were then incubated with a secondary antibody conjugated with horseradish peroxidase (dilution 1 : 10000) and developed using the ECL detection system. The immunoreactive bands were visualised by autoradiography and the density of the bands was evaluated densitometrically using Gel-Pro Analyzer 4.5, 2000 software (Media Cybernetics, Silver Spring, MD, USA). The membranes were stripped and incubated with tubulin monoclonal antibody (dilution 1 : 5000) and subsequently with an anti-mouse antibody (dilution 1 : 10000) to assess gel-loading homogeneity.

### 2.8. Immunohistochemistry

Immunohistochemical staining was performed on 10-*μ*m acetone fixed cryostatic sections of gastrocnemius. Nonspecific binding sites were blocked for 1 h with 3% BSA in PBS. For immunodetection of glucose transporter type-4 (GLUT-4), sections were incubated overnight with rabbit anti-GLUT-4 antibody (Abcam), dilution 1 : 200, and for 1 hour with goat anti-rabbit IgG-HRP conjugated secondary antibody (Bio-Rad Laboratories, Hercules, CA, USA). For detection of glucose transporter type 5 (GLUT-5), sections were incubated overnight with rabbit anti-GLUT-5 antibody (Abcam), dilution 1 : 50, for 1 hour with swine anti-rabbit IgG-biotinylated secondary antibody (Dako, Glostrup, Denmark), and for 1 hour with Streptavidin HRP conjugate (Southern Biotech, Birmingham, AL, USA). The specific staining was detected with diaminobenzidine (DAB, Sigma-Aldrich), and sections were visualized with Olympus-Bx4I microscope connected by a photographic attachment (Carl Zeiss, Oberkochen, Germany). For each antibody, a negative control was included in which the primary antibody was replaced with a nonimmune isotypic control antibody.

### 2.9. Determination of IL-6 and FGF-21

Serum levels of IL-6 and FGF-21 levels in gastrocnemius homogenates were measured by ELISA according to the manufacturer's instructions (Mouse FGF-21 and IL-6 ELISA kits, R&D Systems, Abingdon, UK).

### 2.10. Materials

Unless otherwise stated, all compounds were purchased from the Sigma-Aldrich Company Ltd. (St. Louis, MO, USA). The BCA Protein Assay kit and SuperBlock blocking buffer were from Pierce Biotechnology Inc. (Rockford, IL, USA), and PVDF was from the Millipore Corporation (Bedford, Massachusetts, USA). Antibodies were from Cell-Signaling Technology (Beverly, MA, USA), Santa Cruz Biotechnology (Santa Cruz, CA, USA), and Abcam (Cambridge, CB, UK). The anti-mouse, anti-rabbit, and anti-goat horseradish peroxidase-linked antibodies were from Santa Cruz Biotechnology (Santa Cruz, CA, USA), and Luminol ECL was from PerkinElmer (Waltham Massachusetts, USA). 

### 2.11. Statistical Analysis

All values in both the text and figures are expressed as mean ± SD for *n* observations. One-way analysis of variance with Dunnett's post-hoc test was performed using the GraphPad Prism version 4.02 for Windows (GraphPad Software, San Diego, California, USA), and *P* values below 0.05 were considered as significant.

## 3. Results

### 3.1. Effects of HFCS Diet and GW0742 Administration on Body Mass Change, Food Intake, and Blood Parameters

After 30 weeks of dietary manipulation, mice on HFCS group had significantly higher body mass than the control group, with an increase in body mass over 30% (36.00 ± 1.41 g versus 32.25 ± 1.67 g; *P* < 0.01), whereas GW0742 administration induced a slight but not significant reduction in body mass (35.00 ± 2.83 g). Epididymal fat mass was increased by HFCS manipulation in comparison to control diet (4.22 ± 0.35% body mass versus 3.66 ± 0.29% body mass; *P* < 0.01), and values were reduced to the control level by drug administration (3.86 ± 0.33% body mass). In contrast, neither dietary manipulation nor drug treatment affected gastrocnemius mass (% body mass: 0.97 ± 0.19 control, 0.92 ± 0.09 HFCS, and 0.92 ± 0.06  HFCS + GW). As we previously reported [5], the HFCS diet caused a significant increase in serum triglycerides, total cholesterol, and LDL concentrations and a marked decrease in the HDL levels. GW0742 administration reverted the deleterious effects of HFCS diet on the serum lipid profile. Interestingly, the HFCS diet significantly affected insulin sensitivity. A significant increase in serum insulin levels was observed in mice of HFCS group compared to control mice (2.26 ± 0.51 *μ*g/L versus 1.27 ± 0.07 *μ*g/L), and this increase was almost completely abolished by GW0742 (1.51 ± 0.46 *μ*g/L). Fasting glucose concentrations were elevated in serum of HFCS animals in comparison to control animals (103.4 ± 12.0 mg/dL versus 78.7 ± 0.3 mg/dL) and reduced to control levels by GW0742 (81.3 ± 10.9 mg/dL). Moreover, HFCS mice showed a significant impairment in glucose tolerance to exogenously administered glucose. Although all the groups reached a glycemic peak at 15 min postglucose challenge (control: 223.1 ± 12.0 mg/dL; HFCS: 212.7 ± 7.3 mg/dL; HFCS + GW: 198.8 ± 10.3 mg/dL), glycemic levels at 30–60 min post glucose challenge in HFCS-fed mice were higher than those recorded in the control group (30 min: 182.9 ± 8.3 mg/dL versus 129.4 ± 8.0 mg/dL, *P* < 0.05; 60 min: 130.7 ± 10.3 mg/dL versus 96.8 ± 4.0 mg/dL, *P* < 0.05). GW0742 significantly (*P* < 0.05) improved glucose tolerance in HFCS-fed mice, showing significantly reduced glucose concentrations at both 30 and 60 min postglucose challenge (89.7 ± 5.2 mg/dL and 86.7 ± 4.3 mg/dL, resp.).

Data on the effects of dietary manipulation and chronic GW0742 treatment on serum levels of IL-6, adiponectin, and leptin are reported in Table 1. The experimental diet caused a more than fourfold increase in serum IL-6 concentrations, whereas GW0742 administration significantly decreased the IL-6 levels. Adiponectin and leptin showed different patterns: in fact, the level of adiponectin in HFCS mice was lower than in control mice, while that of leptin increased versus controls. When GW0742 was administered to HFCS mice there was a significant increase (*P* < 0.01) in the adiponectin levels associated with a significant decrease (*P* < 0.01) in leptin serum concentration. 

It must be noted that in control mice GW0742 had no significant effect on any of the previously described metabolic parameters.

### 3.2. Effect of GW0742 on Skeletal Muscle PPAR-*δ* Expression

**Figure 1:**
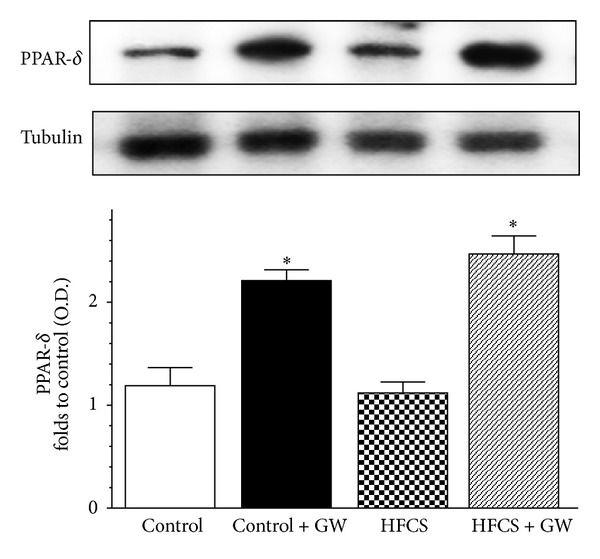
Effects of dietary manipulation and GW0742 treatment on PPAR-*δ* expression in the mouse gastrocnemius. Protein expression was measured by western blot analysis in gastrocnemius homogenates of mice fed with a standard (control) or HFCS diet (HFCS) in the absence or presence of GW0742 treatment (1 mg/kg/day) (control + GW; HFCS + GW). Densitometric analysis of the bands is expressed as relative optical density (O.D.), corrected for the corresponding tubulin, and normalized using the related control band. Data are means ± SD of three separate experiments. *P < 0.01 versus HFCS.

As shown in Figure 1, HFCS diet did not affect PPAR-*δ* protein level expression in the mouse gastrocnemius. In contrast, daily administration of GW0742 resulted in a twofold increase in expression of its pharmacological target, with maximum effect in the presence of dietary manipulation. 

### 3.3. The Effects of HFCS Diet on Insulin Signal Transduction Were Reverted by GW0742 Administration

**Figure 2:**
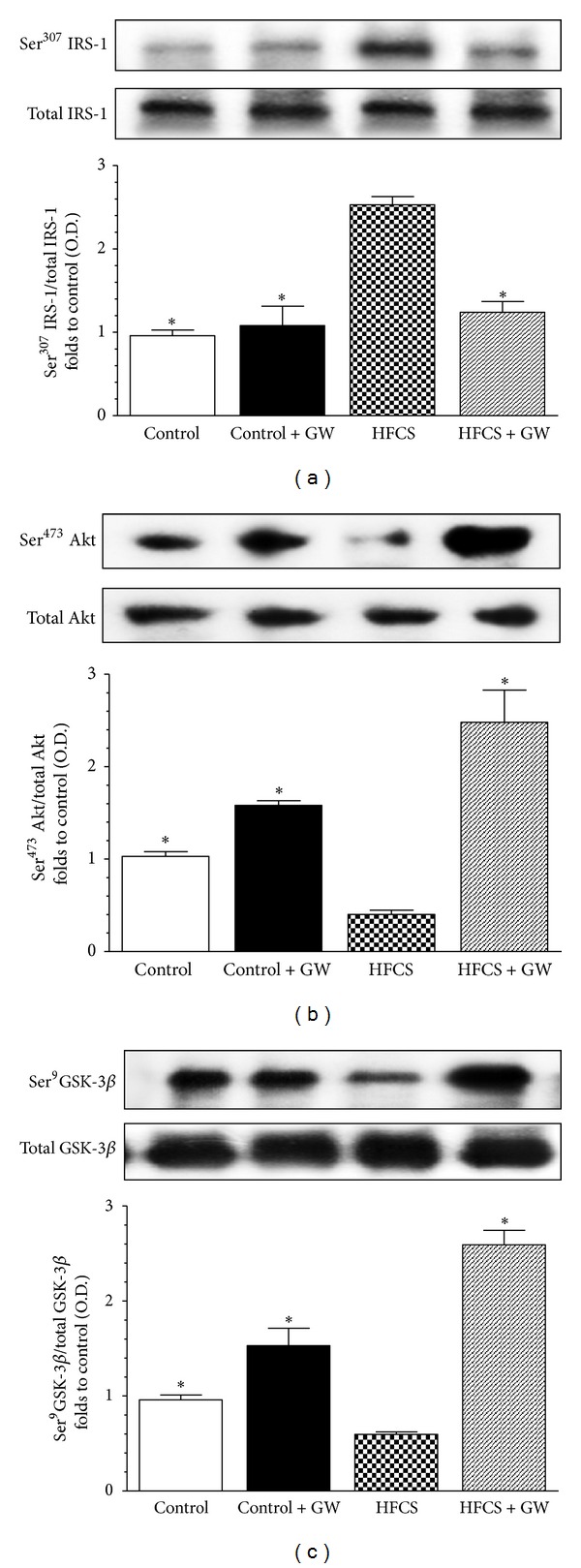
Effects of GW0742 treatment on insulin signal transduction in the gastrocnemius of mice fed with HFCS diet. Total IRS-1 protein expression and Ser^307^ phosphorylation (a), total Akt protein expression and Ser^473^ phosphorylation (b), and total GSK-3*β* protein expression and Ser^9^ phosphorylation (c) were analyzed by western blot on the gastrocnemius homogenates obtained from mice fed with a standard (control) or HFCS diet (HFCS) for 30 weeks and treated with GW0742 (1 mg/kg/day) added during the last 16 weeks (control + GW; HFCS + GW). Densitometric analysis of the bands is expressed as relative optical density (O.D.), corrected for the corresponding tubulin contents, and normalized using the related control band. The data are means ± SD of three separate experiments. *P < 0.01 versus HFCS.

The HFCS diet did not alter the protein expression of IRS-1, Akt, or GSK-3*β* compared to the control group. However, HFCS caused a marked increase in Ser^307^ phosphorylation of IRS-1 in parallel with reduced Ser^473^ phosphorylation of Akt (Figures 2(a) and 2(b)). Ser^9^ phosphorylation of GSK-3*β*, a downstream target of Akt, was also reduced in the presence of HFCS (Figure 2(c)), suggestive of impaired insulin signaling downstream of IRS-1. Most notably, GW0742 significantly attenuated all the effects of HFCS on IRS-1, Akt, and GSK-3*β* phosphorylation, measured at the steady state.

### 3.4. Effects of HFCS Diet and GW0742 Treatment on GLUT-4 and GLUT-5 Expression and Translocation

**Figure 3:**
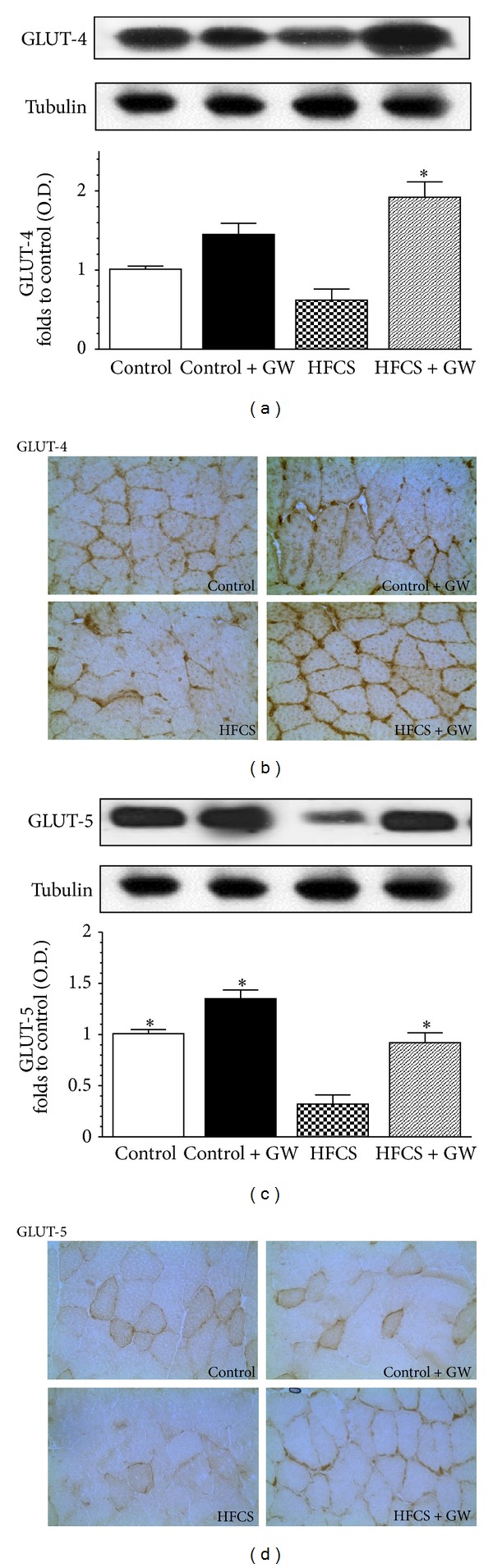
Effects of two dietary regimens either normal (control) or a diet enriched with 15% HFCS solution (HFCS) on GLUT-4 and GLUT-5 expression (resp., (a) and (c)) and membrane translocation (resp., (b) and (d), original magnification: 400x) in the gastrocnemius of mice treated with GW0742 (1 mg/kg/day, control + GW; HFCS + GW). Densitometric analysis of the bands is expressed as relative optical density (O.D.), corrected for the corresponding tubulin, and normalized using the related control band. Data are means ± SD of three separate experiments. *P < 0.01 versus HFCS.

In comparison to control animals, GLUT-4 expression was reduced in the gastrocnemius of HFCS mice, without reaching a statistical significance, and increased following drug treatment (Figure 3(a)). Similarly, the HFCS diet markedly reduced GLUT-5 expression, whereas GW0742 administration resulted in a significant increase in GLUT-5 expression (Figure 3(c)). Notably, GW0742 treatment not only increased carriers expression levels but also induced a significant membrane translocation, thus increasing muscle glucose uptake (Figures 3(b) and 3(d)).

### 3.5. Effects of HFCS Diet and GW0742 Treatment on Skeletal Muscle Triglyceride Content and AMPK/ACC and CPT-1 Signaling Pathway

**Figure 4:**
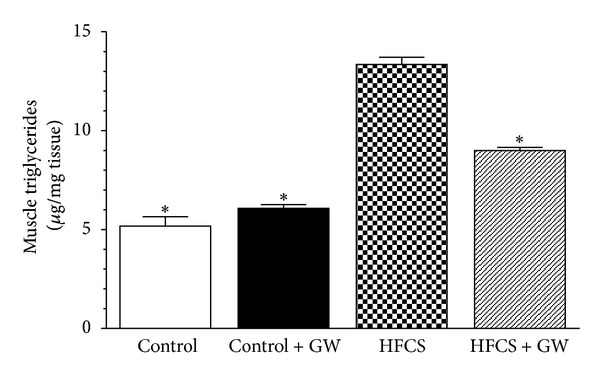
Triglyceride content in gastrocnemius of mice fed with a standard diet (control) or a HFCS diet (HFCS) for 30 weeks and treated with GW0742 (1 mg/kg/day) added during the last 16 weeks (control + GW; HFCS + GW). Data are means ± SD of eight animals/group. *P < 0.01 versus HFCS.

The triglyceride content was doubled in the gastrocnemius of HFCS mice in comparison with control animals, whereas skeletal muscle triglyceride accumulation was significantly reduced by GW0742 administration (Figure 4). 

**Figure 5:**
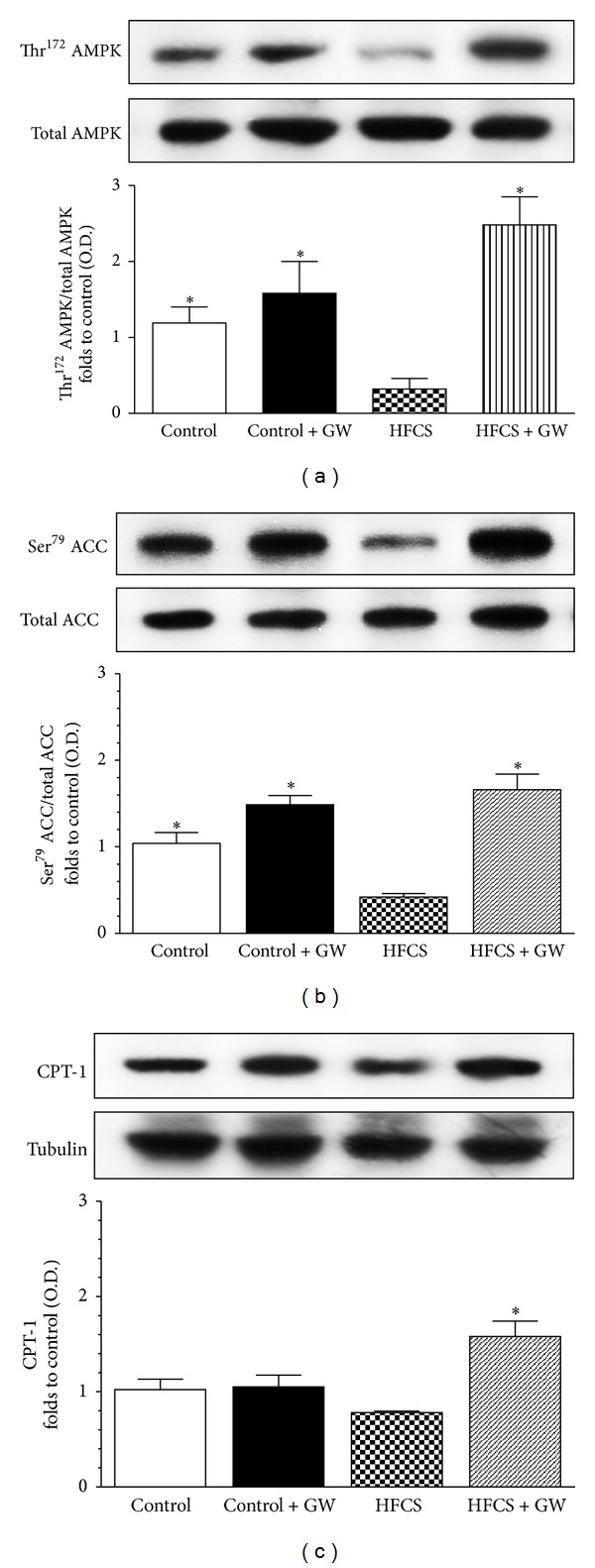
Effects of GW0742 treatment on AMPK/ACC phosphorylation and CPT-1 expression in the gastrocnemius of mice fed with HFCS diet. Total AMPK protein expression and Thr^172^ phosphorylation (a), total ACC protein expression and Ser^79^ phosphorylation (b), and CPT-1 expression (c) were analyzed by western blot on the gastrocnemius homogenates obtained from mice fed with a standard (control) or HFCS diet (HFCS) for 30 weeks and treated with GW0742 (1 mg/kg/day) added during the last 16 weeks (control + GW; HFCS + GW). Densitometric analysis of the bands is expressed as relative optical density (O.D.), corrected for the corresponding tubulin contents, and normalized using the related control band. The data are means ± SD of three separate experiments. *P < 0.01 versus HFCS.

Changes in the phosphorylation/activation of the AMP-activated protein kinase (AMPK)/acetyl-CoA carboxylase (ACC) system, whose central role in the regulation of cellular lipid homeostasis is well known, were evaluated by immunoblotting experiments on gastrocnemius homogenates. As reported in Figure 5, there was no significant effect of HFCS feeding or drug treatment on total AMPK and ACC protein content. In contrast, chronic HFCS exposure markedly reduced Thr^172^ phosphorylation of AMPK and its substrate Ser^79^ ACC. Interestingly, GW0742 treatment led to a robust increase in phosphorylation of both AMPK and ACC in HFCS-fed mice (Figures 5(a) and 5(b)). The effects of PPAR-*δ* agonism on fatty acid oxidation in skeletal muscle cells are further indicated by the significant increase in the protein expression of carnitine palmitoyl transferase-1 (CPT-1) elicited by GW0742 in HFCS-fed mice (Figure 5(c)).

### 3.6. Effects of HFCS Diet and GW0742 Treatment on NF-*κ*B Activation, ICAM-1, and iNOS Expression

**Figure 6:**
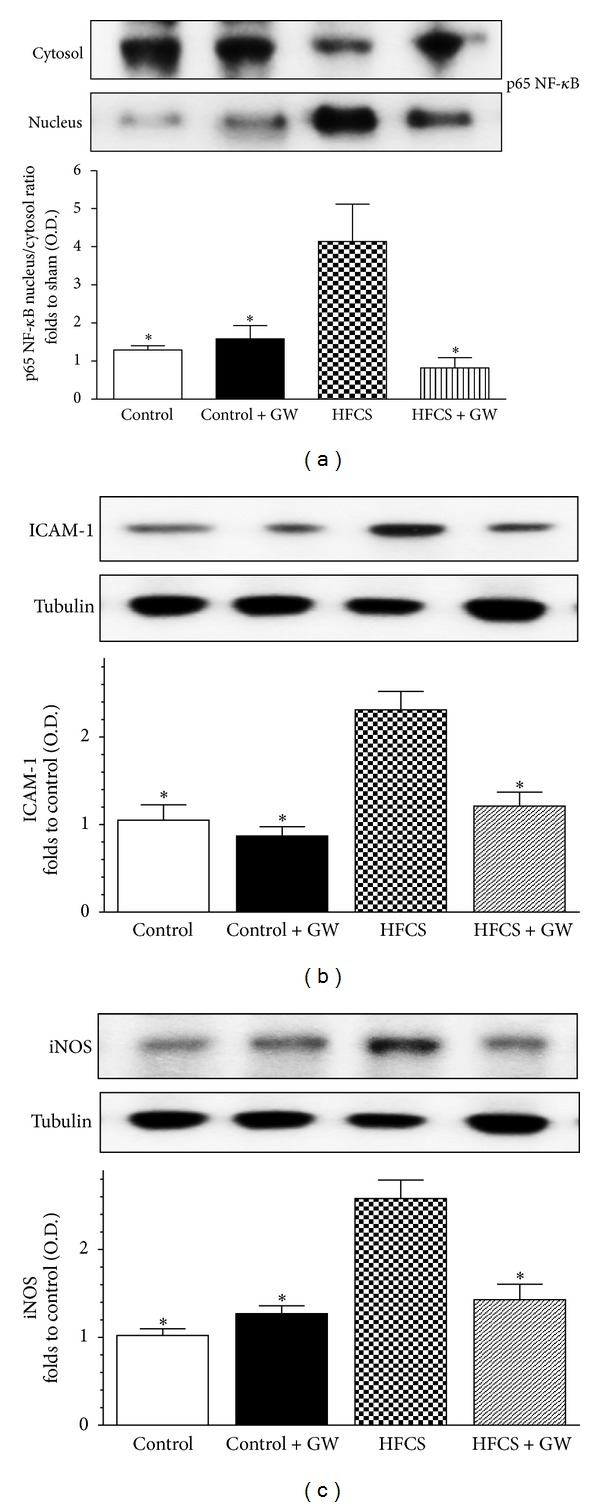
Effects of dietary manipulation and GW0742 treatment on NF-*κ*B p65 translocation (a), ICAM-1 (b) and iNOS (c) protein expression in the mouse gastrocnemius. Protein expression was measured by Western blot analysis in gastrocnemius homogenates of mice fed with a standard (control) or HFCS diet (HFCS) in the absence or presence of GW0742 treatment (1 mg/kg/day) (control + GW; HFCS + GW). Densitometric analysis of the bands is expressed as relative optical density (O.D.), corrected for the corresponding tubulin contents, and normalized using the related control band. NF-*κ*B p65 subunit levels in cytosolic and nuclear fractions are expressed as nucleus/cytosol ratio normalized using the related control band. Data are means ± SD of three separate experiments. *P < 0.01 versus HFCS.

To investigate the muscle inflammatory response induced by HFCS-diet and the intracellular signaling pathway(s) that might be involved in the protective mechanisms evoked by GW0742, we firstly evaluated the effects on NF-*κ*B activation. When compared to control mice, HFCS mice developed significant increase in the nuclear translocation of the p65 NF-*κ*B subunit in the mouse gastrocnemius, indicating the activation of this transcriptional factor (Figure 6(a)). Treatment with GW0742 resulted in a significant reduction in nuclear translocation of p65 and, hence, in the activation of NF-*κ*B. The intercellular adhesion molecule-1 (ICAM-1), whose role in the recruitment of neutrophils is widely accepted, was slightly detected in the gastrocnemius of control animals, whereas its expression was dramatically increased by HFCS diet (Figure 6(b)). As shown in Figure 6(c), also the expression of the inducible nitric oxide synthase (iNOS) was increased in the presence of dietary manipulation. Interestingly, these changes were suppressed by GW0742 administration, as evidenced by densitometric analysis of the related autoradiograms.

### 3.7. Effects of HFCS Diet and GW0742 Treatment on Skeletal Muscle FGF-21 Production

**Figure 7:**
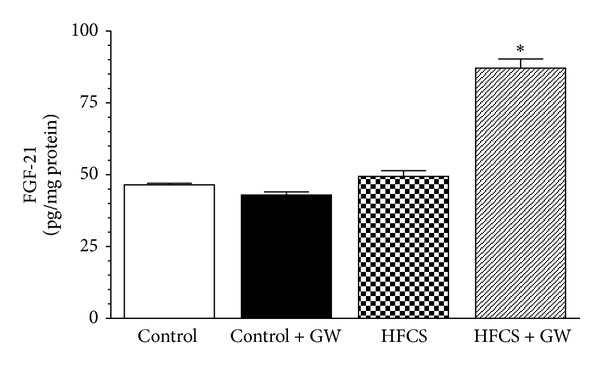
Fibroblast growth factor-21 (FGF-21) levels were analyzed by ELISA in gastrocnemius homogenates of mice fed with a standard (control) or HFCS diet in the absence or presence of GW0742 (1 mg/kg/day). Data are means ± SD of five animals/group. *P < 0.01 versus HFCS.

The beneficial effects of PPAR-*δ* activation were associated with a dramatic increase of FGF-21 production in the mouse gastrocnemius. As shown in Figure 7, in comparison to control mice (46.45 ± 1.13 pg/mg protein), HFCS diet did not affect the production of this myokine (49.38 ± 4.12 pg/mg protein), whereas GW0742 treatment induced a twofold increase (87.08 ± 6.37 pg/mg protein). 

## 4. Discussion

In agreement with previous observations [14, 15], the present study shows that chronic exposure to the most widely used added sugar HFCS caused a significant increase in body mass associated with increases in serum levels of triglycerides, LDL-cholesterol, glucose, insulin, IL-6, leptin, and hypoadiponectinemia. Using the same experimental protocol here reported, we have recently demonstrated that the molecular mechanism underlying the deleterious effects of HFCS involves the hepatic upregulation of fructokinase, the main fructose-metabolizing enzyme, which may account for the increase in serum levels of free fatty acids and hyperuricemia [5]. We also reported that the documented increase in serum uric acid level significantly contributed to the development of chronic kidney injury. Interestingly, we demonstrated that chronic administration of the PPAR-*δ* ligand GW0742 exerted beneficial effects by preventing the upregulation of fructokinase in the liver and activation of the inflammatory signaling complex NLRP3 inflammasome in the kidney. Despite these preliminary data, so far, there are no studies on the effects of long-term exposure to HFCS, the major sweetener added to beverages and food, on skeletal muscle, which is a major site of postprandial glucose disposal and is therefore one of the insulin-sensitive tissues most likely to manifest early signs of insulin resistance. Here, we investigated (i) the mechanisms underlying metabolic disturbances in skeletal muscle of mice exposed for 30 weeks to high intake of HFCS and (ii) the local effects evoked by PPAR-*δ* chronic activation. The sugar was added to the drink water at a concentration that covers 10% of the daily caloric intake, corresponding to the average energy intake in the form of ingested sweeteners in the western diet. We documented the HFCS-induced alteration in the insulin signal transduction pathway, as shown by the impaired phosphorylation of IRS-1 protein as well as of the downstream key insulin signaling molecules, Akt, and GSK-3*β*, an Akt substrate [16]. This is in keeping with previous studies showing that IRS-1 serine phosphorylation can interfere with subsequent Akt and GSK-3*β* phosphorylation, disrupting insulin signal transduction [17]. In our experimental model, oral administration of the selective PPAR-*δ* agonist GW0742 was associated with a significant improvement of the defective insulin signaling in the skeletal muscle, which may account at least in part for the changes in serum lipid profile and insulin sensitivity. As the inhibition of GSK-3*β* evokes an increase in the glycogen synthesis [18, 19], we may speculate that GW0742 administration modulates muscle glycogen storage through inactivation of GSK-3*β*. Although no direct evidence of drug treatment on glucose storage has been reported in our study, we documented the effects of chronic PPAR-*δ* activation on glucose transporters expression and distribution. Specifically, we found that PPAR-*δ* activation by GW0742 evoked an increase in expression of GLUT-4, the most abundant glucose transporter isoform in skeletal muscle [20], and its translocation from intracellular compartments to the plasma membrane, thus facilitating glucose transport. Similarly, GLUT-5, the fructose carrier with low capacity to transport glucose [21], was scarcely detectable in the gastrocnemius muscle of HFCS-fed mice, and its expression and membrane translocation were enhanced by chronic PPAR-*δ* activation. Because fructose is highly present in the HFCS diet and contributes to carbohydrate metabolism in muscle [22], the increase in GLUT-5 may favor a better utilization of fructose supplied in the diet. In any case, the reduction in GLUT-4 and GLUT-5 expression and translocation detected in the HFCS group could represent an adaptation to the chronic exposure to a sugar-enriched diet, while the increases in these carrier isoforms in the presence of the selective PPAR-*δ* agonist GW0742 may account, at least in part, for the improvement in insulin-induced muscle glucose uptake. Recent data have shown that Akt inhibitors or dominant-negative Akt constructs regulate the translocation, targeting, and fusion of GLUT-4-containing vesicles [23, 24]. Similarly, IRS-1 phosphorylation has been reported to affect GLUT-4 translocation and subsequent glucose uptake in mouse skeletal myocytes [25]. We may, thus, speculate that the beneficial effects of the PPAR-*δ* ligand GW0742 are secondary to activation of the IRS-1/Akt/GSK-3*β* pathway. This is also supported by previous findings showing that the chronic administration of GW0742 in rats results in phosphoinositide-dependent kinase phosphorylation and, hence, increased Akt phosphorylation [26]. Another enzyme complex which could be involved in PPAR-*δ* metabolic effects within the skeletal muscle is AMPK, a serine/threonine protein kinase that has emerged as a key player in both lipid and glucose metabolism in skeletal muscles [27, 28]. In mouse C2C12 myotubes, AMPK activation potentiated insulin action by reducing IRS-1 serine phosphorylation [29], while reduced muscle AMPK activity has been reported to aggravate muscle insulin resistance following a high-fat diet over 30 weeks [30]. In agreement with these observations, we found that GW0742 increased phosphorylation of Thr^172^ regulatory site on AMPK which, in turn, causes ACC phosphorylation. When ACC is inactive (phosphorylated form), a fall in malonyl-coenzyme A occurs, which disinhibits CPT-1 and increases mitochondrial import and oxidation of long-chain fatty acids [31]. Our data demonstrate that chronic exposure to GW0742 administration increased skeletal muscle CPT-1 expression, thus shunting toward fatty acid oxidation, and this effect was associated with reduced triglyceride accumulation. These findings suggest that the beneficial effects evoked by the PPAR-*δ* agonist GW0742 are at least partially dependent on AMPK activation. The direct involvement of PPAR-*δ* agonism in mediating improvements in muscle insulin sensitivity and lipid metabolism is also confirmed by results showing that PPAR-*δ* is expressed in the gastrocnemius of both control and HFCS-fed mice and, more importantly, that its expression is substantially increased by chronic administration of GW0742, thus suggesting that the observed drug effects are due to a ligand-dependent PPAR-*δ* activation. This is consistent with previous works showing that PPAR-*δ* activation is involved in regulating myogenesis [32–35]. In search of the mechanism(s) underlying the protective action of GW0742, we investigated whether PPAR-*δ* activation may affect the local inflammatory response associated with muscular metabolic injury. Although PPAR-*δ* has been implicated in the regulation of systemic inflammatory responses associated with metabolic dysregulation [7], so far, reports on anti-inflammatory effects of PPAR activation in skeletal muscle are rather scarce. Here, we show for the first time that in the skeletal muscle GW0742 reverts the diet-induced increase in the nuclear translocation of NF-*κ*B p65, a transcriptional factor that plays an important role in regulating the transcription of a number of genes, especially those involved in producing mediators of local and systemic inflammation (such as cytokines, chemokines, and cell adhesion molecules). This observation is in agreement with previous studies reporting that PPAR-*δ* activation induces the physical interaction between PPAR-*δ* and the p65 subunit of NF-*κ*B and reduced the LPS-induced degradation of the inhibitory protein “Inhibitor of kappa B,” thus preventing NF-*κ*B activation [36, 37]. The reduction of NF-*κ*B activation by GW0742 treatment may account for the observed reduction in the serum levels of IL-6, which is known to be mostly released from skeletal muscle, and in the local expression of the NF-*κ*B-dependent proteins iNOS and ICAM-1, whose role in the development of insulin resistance has been recently documented [38, 39]. As several studies have reported an association between GSK-3*β* and NF-*κ*B activity [40], we might speculate that PPAR-*δ* activation phosphorylates, and hence, activates the Akt pathway, which in turn phosphorylates, and hence, inhibits GSK-3*β*, presumably resulting in the inhibition of NF-*κ*B and, in turn, NF-*κ*B-dependent proinflammatory gene transcription. Although this cross talk among different signaling pathways by pharmacological PPAR-*δ* modulation is intriguing, it must be stressed, however, that the lack of direct experimental evidence of a causal relationship between the improved local and systemic insulin sensitivity and the treatment-induced reduction of NF-*κ*B activation in our experimental model limit the interpretation of the molecular mechanism(s) underlying our findings. One of the most recently identified mediators that facilitates organ cross talk and the related control of impaired glucose homeostasis in metabolic diseases is a member of the FGF family, FGF-21. FGF-21 has been previously shown to lower blood glucose levels in several diabetic rodent and monkey models [41, 42], to regulate lypolisis in white adipose tissue [43, 44] and substrate utilization in the liver [45]. FGF-21 was also shown to directly enhance skeletal muscle glucose uptake [46]. The metabolic effects of FGF-21 on glucose metabolism involve selective regulation by different PPAR isoforms. Liver-derived FGF-21 is stimulated by PPAR-*α* ligands [47]. Conversely, PPAR-*γ* activation with feeding promotes FGF-21 production in adipocytes but not in liver [48, 49]. To our knowledge, this is the first paper that demonstrates a correlation between PPAR-*δ* activation and increased FGF-21 levels in skeletal muscle, thus adding an original piece of evidence to the complex mechanisms by which PPAR-*δ* can regulate several biological functions. Our observation is also in keeping with a previous study showing that circulating FGF-21 levels consistently increase in human subjects in response to pharmacological activation of PPAR-*δ* [50]. Although a previous investigation has revealed that PPAR-*γ* directly regulates expression of the FGF-21 gene through elements located within the 500-bp upstream region of the gene [49], to date, a consensus sequence of PPAR-*δ* binding site in the promoter region of the FGF-21 gene has not yet been identified. Besides *in vitro* experiments with FGF-21, small interfering RNA are warranted to clarify whether GW0742 beneficial effects are related to the direct modulation of FGF-21 biological functions. The recent findings on FGF-21 ability to prevent insulin resistance in human myoblasts by inhibiting NF-*κ*B activation [51] may also suggest that the muscle-derived FGF-21 acts in an autocrine fashion to amplify PPAR-*δ* inhibitory effects on the expression of NF-*κ*B-dependent inflammatory genes. This further supports the existence of multiple anti-inflammatory pathways involved in the beneficial effects evoked by PPAR-*δ* activation. 

In conclusion, we have shown that feeding mice with a HFCS diet for 30 weeks evoked skeletal muscle insulin resistance and lipid accumulation which were associated with activation of inflammatory pathways. All these effects were attenuated by selective PPAR-*δ* activation. In addition, this study, showing a strong induction of FGF-21 in the skeletal muscle after chronic administration of the PPAR-*δ* ligand GW0742, enhances our knowledge of the mechanisms of action of PPAR-*δ* agonism and provides further insights into the role of FGF-21 as mediator of the tissue cross-talk that underlines the integrated control of the metabolic inflammation.

## Figures and Tables

**Table 1:** Effects of chronic *in vivo *treatment with GW0742 on mouse blood parameters after 30 weeks of dietary manipulation.

	Control (*n* = 10)	Control + GW0742 (*n* = 6)	HFCS (*n* = 10)	HFCS + GW0742 (*n* = 10)
IL-6 (pg/mL)	20.80 ± 2.11	18.90 ± 4.22	91.55 ± 16.47*	28.80 ± 7.99^§^
Adiponectin (*µ*g/mL)	4.53 ± 0.23	4.75 ± 0.46	1.93 ± 0.08*	3.69 ± 0.43^∗§^
Leptin (pg/mL)	206.65 ± 18.77	198.07 ± 22.17	446.67 ± 52.61*	266.12 ± 41.21^∗§^

HFCS: high-fructose corn syrup; IL-6: interleukin-6.

Data are means ± S.D.

*P < 0.01 versus Control.

^§^ *P* < 0.01 versus HFCS.
